# eHealth and mHealth Psychosocial Interventions for Youths With Chronic Illnesses: Systematic Review

**DOI:** 10.2196/22329

**Published:** 2020-11-10

**Authors:** Nancy Lau, Shayna Waldbaum, Ryan Parigoris, Alison O'Daffer, Casey Walsh, Susannah F Colt, Joyce P Yi-Frazier, Tonya M Palermo, Elizabeth McCauley, Abby R Rosenberg

**Affiliations:** 1 Palliative Care and Resilience Lab Center for Clinical and Translational Research Seattle Children's Research Institute Seattle, WA United States; 2 Department of Psychiatry and Behavioral Sciences University of Washington School of Medicine Seattle, WA United States; 3 Chicago Medical School Rosalind Franklin University of Medicine and Science North Chicago, IL United States; 4 Department of Psychology University of Massachusetts Boston Boston, MA United States; 5 Department of Health Services University of Washington Seattle, WA United States; 6 Clinical Research Division Fred Hutchinson Cancer Research Center Seattle, WA United States; 7 Perelman School of Medicine University of Pennsylvania Philadelphia, PA United States; 8 Department of Anesthesiology and Pain Medicine University of Washington School of Medicine Seattle, WA United States; 9 Center for Child Health, Behavior, and Development Seattle Children's Research Institute Seattle, WA United States; 10 Cambia Palliative Care Center of Excellence University of Washington Seattle, WA United States; 11 Department of Pediatrics University of Washington School of Medicine Seattle, WA United States

**Keywords:** pediatrics, chronic illness, mHealth, eHealth, psychosocial interventions, mental health

## Abstract

**Background:**

An estimated 12.8% of children and adolescents experience chronic health conditions that lead to poor quality of life, adjustment and coping issues, and concurrent mental health problems. Digital health deployment of psychosocial interventions to support youth with chronic illness has become increasingly popular with the advent of the technological advances in the digital age.

**Objective:**

Our objectives were to systematically review published efficacy studies of eHealth and mHealth (mobile health) psychosocial interventions for youths with chronic illnesses and review intervention theory and treatment components.

**Methods:**

PubMed, Embase, Web of Science, PsycInfo, and Cochrane Database of Systematic Reviews were searched for studies published from 2008 to 2019 of eHealth and mHealth psychosocial interventions designed for children and adolescents with chronic illnesses in which efficacy outcomes were reported. We excluded studies of interventions for caregivers, healthy youth, disease and medication management, and telehealth interventions that function solely as a platform to connect patients to providers via phone, text, or videoconference.

**Results:**

We screened 2551 articles and 133 relevant full-text articles. Sixteen efficacy studies with psychosocial and health outcomes representing 12 unique interventions met the inclusion criteria. Of the included studies, 12 were randomized controlled trials and 4 were prospective cohort studies with no comparison group. Most interventions were based in cognitive behavioral theory and designed as eHealth interventions; only 2 were designed as mHealth interventions. All but 2 interventions provided access to support staff via text, phone, email, or discussion forums. The significant heterogeneity in intervention content, intervention structure, medical diagnoses, and outcomes precluded meta-analysis. For example, measurement time points ranged from immediately postcompletion of the mHealth program to 18 months later, and we identified 39 unique outcomes of interest. The majority of included studies (11/16, 69%) reported significant changes in measured health and/or psychosocial posttreatment outcomes, with small to large effect sizes.

**Conclusions:**

Although the available literature on the efficacy of eHealth and mHealth psychosocial interventions for youth with chronic illnesses is limited, preliminary research suggests some evidence of positive treatment responses. Future studies should continue to evaluate whether digital health platforms may be a viable alternative model of delivery to traditional face-to-face approaches.

## Introduction

An increasing number of youths (ie, children and adolescents aged 18 years and younger) are diagnosed with a chronic condition in the United States, with an estimated prevalence rate of 12.8% [1-3]. Chronic illness in childhood negatively impacts quality of life [4,5]. Chronic health conditions can lead to emotional challenges and heighten coping difficulties [6]. Up to 60% of children with a chronic illness have at least one co-occurring psychological disorder [7], compared with 10% to 20% of the general pediatric population [8]. Across illness type, stressors associated with chronic disease are vastly similar. Burdens to these populations include treatment-related stress, changes to daily life and routines, and uncertainty about the future [7,9].

Face-to-face psychosocial interventions such as cognitive behavioral therapy are designed to teach and bolster coping skills and improve psychological adjustment [10]. Such interventions have been developed for various illness populations to improve psychosocial outcomes and quality of life [11]. However, barriers to in-person treatment include limited availability of and access to psychosocial clinicians and high costs of treatment [12,13]. A majority of youths screened in school and primary care settings with elevated mental health symptoms do not follow-up with referrals to mental health clinicians, especially those who are racial/ethnic minorities, have public insurance, or come from low-income households [14]. Additionally, there are workforce shortages in proportion to demand and need, with wait times for psychiatric care appointments exceeding that of pediatricians [15]. A potential solution is leveraging technological advances and digital media to deploy behavioral health interventions on a larger scale. Internet-based interventions (otherwise known as eHealth interventions) confer the advantages of instant availability, anonymity, self-pacing, the ability to reach patients in remote areas, and cost-effectiveness due to reduced personnel and infrastructure requirements [16,17].

Internet-based interventions may be particularly appealing to younger generations who are digital natives accustomed to interacting on smartphones and the internet [16-18]. The internet serves as a primary means of health-related and mental health–related information-seeking and communication for youths [19,20]. Additionally, young people endorse reluctance to seek psychological services due to social stigma, discomfort discussing personal problems, and a preference for self-help [16,17,19,21]. Thus, there has been a rapid growth in the use of eHealth platforms to deploy skills-based behavioral health programs for youths. Moreover, in recent years with the increased use of smartphones there has been a corresponding increase in mobile health (mHealth) apps for symptom self-management on smartphone devices [22,23].

Previous systematic reviews have examined digital interventions in pediatric populations for disease self-management and alleviation of mental health symptoms. Examples include remote management of pediatric chronic pain [24] and technological interventions for asthma self-management in children and adolescents [25-27]. Other reviews have focused on digital health interventions for youth mental health problems [28,29] and internet-based cognitive behavioral therapy for children and adolescents [17,30]. The literature has addressed the benefits of eHealth interventions for anxiety and depression [31], technological tools for disease self-management [32], and technology-based family interventions for improving family functioning [33].

To our knowledge, no existing systematic review has been conducted to critically review the literature on eHealth and mHealth psychosocial interventions for youths with chronic illnesses. Previous reviews in this topic area have focused more narrowly on specific chronic conditions, constellations of mental health symptoms, or types of psychotherapy. Our study objectives were to systematically review the efficacy of eHealth and mHealth psychosocial interventions for youths with chronic illnesses and review underlying intervention theory and treatment components.

## Methods

### Literature Search

The search was executed by a research librarian in five databases for articles published from 2008 to 2019: PubMed/MEDLINE, Embase, Web of Science, PsycINFO, and Cochrane Database of Systematic Reviews. We used keywords and Boolean operators [34] to identify original articles on eHealth and mHealth psychosocial interventions designed for youths or young adults with chronic illnesses. Inclusion criteria were (1) available in English; (2) published in peer-reviewed journal; (3) experimental, quasi-experimental, and observational studies in which efficacy outcome(s) were reported; (4) eHealth or mHealth psychosocial interventions (with technology as the primary mode of content delivery, either entirely self-guided or human-assisted); and (5) designed for children and adolescents aged 18 years and younger with chronic disease (ie, a long-term medical condition lasting 3 months or longer [35]).

The original primary search strategy with generic chronic illness search terms is shown in Multimedia Appendix 1. Cancer is a specific condition of interest for our research group for which we were aware of existing digital intervention literature. Based on our primary search strategy, the authors identified several known published studies on digital interventions related to 10 specific chronic illnesses. To ensure all relevant articles were captured, a supplemental search strategy related to the specific chronic illnesses identified was then conducted to generate systematic reviews to search some of the more advanced digital intervention science in pediatrics (Multimedia Appendix 2). Our search strategy was guided and conducted by a medical librarian with extensive experience with systematic reviews; the list of search terms for specific chronic illnesses was modeled after published Cochrane reviews (eg, Law et al [36] on caregiver interventions for children with chronic illness). We excluded studies of interventions that target caregivers or health care providers only, interventions that target mental health problems/disorders not in the context of a chronic medical condition, prevention programs for healthy controls, disease and medication management programs, and programs in which the telehealth platform is only used to connect patients to providers via phone, text, or videoconference.

### Selection of Studies

First, we screened titles and abstracts of studies retrieved for inclusion and exclusion. We then obtained full texts of articles designated as potentially meeting inclusion criteria to assess for eligibility. Screening of all titles, abstracts, and full-text articles was first independently double-coded by authors in pairs (NL, SW; NL, RP; NL, SFC); each dyad coded a subset of articles and NL coded all articles. Then, disagreements between the authors in each dyad were resolved through discussion while referencing the original source material to reach consensus. Finally, for articles meeting inclusion criteria, we independently double-coded relevant information from each study in pairs (NL, SW; NL, RP; NL, SFC), including study design, sample size, target illness, intervention characteristics (eg, intervention theory and components, eHealth or mHealth platform), and treatment outcomes data.

For intervention characteristics, we relied on authors’ descriptions, either provided in the articles themselves or in prior publications of the intervention referenced in the included articles, and standard norms for psychosocial interventions. For example, if authors described an intervention as being based on cognitive behavioral theory, we coded the theory as cognitive behavioral; if the intervention followed a prespecified order mirroring the stepwise progression of traditional manualized evidence-based psychotherapies, it was coded as a modular treatment session per clinical norms [37]; and if patients could connect with research or psychosocial staff for support, we coded the intervention as human-assisted.

Unsurprisingly, measures collected in studies incorporated both psychosocial outcomes and physical health/disease-related outcomes. In the context of chronic medical conditions, physical and psychological consequences are intertwined and physical health/disease-related outcomes tend to improve alongside emotional and psychological functioning [38]. Outcome measures were categorized as either psychosocial (depression, social problem solving, fear and worry about symptoms, anxiety sensitivity, perceived stress, rewarding pain behavior, quality of life, social acceptance, family conflict, pain catastrophizing, psychological well-being, emotional functioning, parental protectiveness, anxiety, school attendance, self-efficacy, posttraumatic stress symptoms, somatic symptoms, coping strategies) or physical health/disease-related (fatigue, physical functioning, energy, disease symptoms, pain intensity and frequency, pain interference, pain reactivity, sleep, disease knowledge, activity limitations, functional disability). Categorizations were based on the psychosocial background literature [39-41] and agreed upon internally by our interdisciplinary research team which includes intervention science researchers, health services researchers, physicians, and psychologists; we acknowledge that some outcomes such as aspects of pain management, sleep, and functional impairments may fit either categorization. For study design, we used author designations. For example, a study was categorized as a pilot randomized controlled trial (RCT) if described as such in the article. We referred to the articles to resolve any discrepancies during consensus conversations and did not make inferences beyond authors’ definitions and descriptions.

After review of the articles, the team determined that heterogeneity in outcome variables and measurement time points precluded meta-analysis. Thus, we described the data systematically.

### Quality Assessment

We independently assessed study quality in pairs (NL, SW; NL, RP; NL, SFC) using the Cochrane Collaboration’s tool for assessing risk of bias [42] to evaluate random sequence generation (selection bias), allocation concealment (selection bias), blinding of participants and personnel (performance bias), blinding of outcome assessment (detection bias), incomplete outcome data (attrition bias), selective reporting (reporting bias), and other biases. We coded each category as low, high, or unclear risk of bias according to established standards in the Cochrane handbook for systematic reviews of interventions [43]. We resolved discrepancies in coding during regularly scheduled consensus meetings by referring to the journal articles themselves.

## Results

### Literature Search

**Figure 1:**
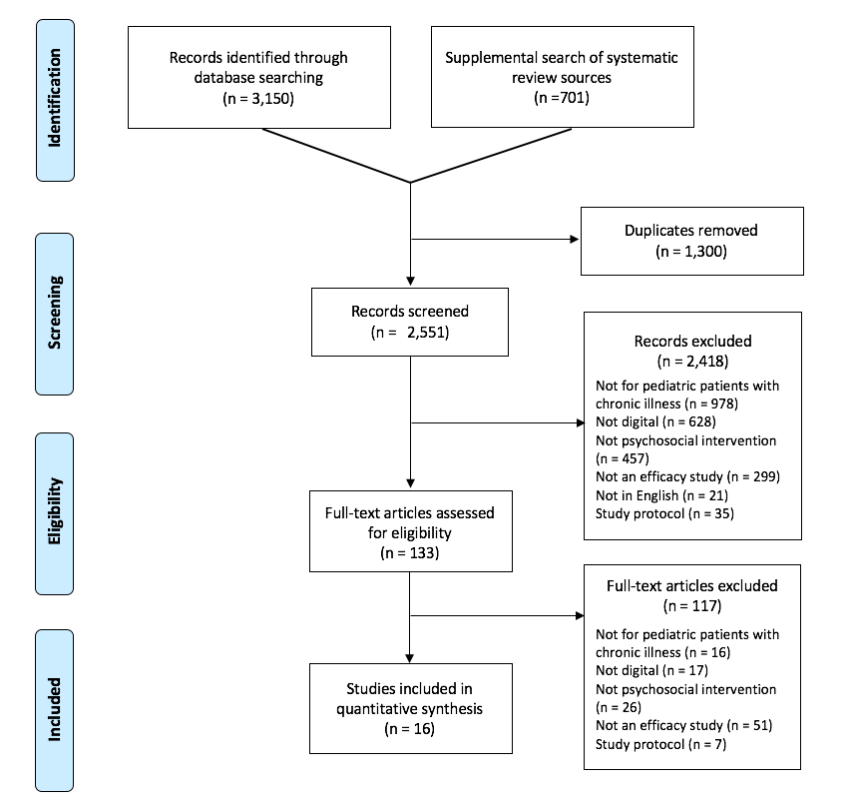
Preferred reporting items for systematic reviews and meta-analyses diagram.

The results of the search and selection of studies are described in the preferred reporting items for systematic reviews and meta-analyses (PRISMA) flow diagram (Figure 1). We screened 2551 articles; 2418 were initially excluded because they did not meet selection criteria. Evaluation of the remaining 133 relevant full-text articles resulted in the exclusion of 117 articles, leaving us with 16 articles that met criteria for inclusion. We provide a synthesis of the findings from the included studies structured around the type of intervention, target population characteristics, intervention content, and type of outcomes.

### Intervention Characteristics

We found 12 [44-60] unique digital interventions that were developed and tested in the United States [51,55,58], Australia [44], Canada [49,60], Germany [48,54], the Netherlands [47], and Sweden [45,61] (Table 1). Ten were eHealth interventions [44-48,51-59,61] and 2 mHealth interventions [49,60]. We found that over half (7/12, 58%) [45-48,53-58,61] were based on cognitive behavioral therapy principles [62,63]. Other treatment frameworks represented included disease self-management, problem solving, psychoeducation, and social-emotional learning. The interventions varied in length, duration, and number of sessions. All but two interventions incorporated modular treatment sessions that follow a prespecified order (Table 1).

**Table 1:** Interventions targeted for youth chronic illness populations.

Intervention name	Targeted illness	Age range^a^	Country of origin	Platform	Standalone intervention or supplement	Iterative design process	Modular or unstructured sessions	Intervention theory
Breathe Easier Online [44]	Chronic respiratory condition	10-17	Australia	eHealth	Standalone		Modular	Evidence-based social support and problem-solving program following PACE^b^ principle
Fatigue in Teenagers on the InterNET [45]	Chronic fatigue syndrome	12-18	Netherlands	eHealth	Standalone		Modular	Evidence-based CBT^c^
iCanCope [60]	Chronic pain	15-18	Canada	mHealth	Standalone	x	Unstructured	Evidence-based psychological pain management, symptom monitoring
iCBT^d^ for adolescents with FGID^e^ [46,61]	Pain-prominent FGID	13-17	Sweden	eHealth	Standalone	x	Modular	Evidence-based iCBT
Move It Now [47]	Chronic pain	12-17	Netherlands	eHealth	Standalone		Modular	Evidence-based CBT
Onco-STEP [48]	Survivors of pediatric cancer	≥15	Germany	eHealth	Standalone		Modular	Evidence-based CBT
PainSquad+ [49,50]	Cancer pain management	12-18	Canada	mHealth	Standalone	x	Unstructured	Evidence-based pharmacological and psychological pain management strategies
TeenCope [51]	Type 1 diabetes	11-14	US	eHealth	Standalone	x	Modular	Evidence-based psychoeducational intervention based on coping skills training
Teens Taking Charge: Managing Arthritis Online [52]	Juvenile idiopathic arthritis	12-18	Canada	eHealth	Standalone	x	Modular	Evidence-based self-management strategies
Trautmann self-help programs: internet-based CBT, internet-based applied relaxation [53,54]	Recurrent headache	10-18^a^	Germany	eHealth	Standalone	x	Modular	Evidence-based CBT, relaxation
Web-based management of adolescent pain [55-57]	Chronic pain, recurrent headache, sickle cell disease	11-18^a^	US	eHealth	Standalone	x	Modular	Evidence-based CBT
Web-based treatment for adolescents with IBD^f^ [58]	Inflammatory bowel disease	12-17	US	eHealth	Supplement to face-to-face	x	Modular	Evidence-based CBT

^a^Age range represents lowest and highest age range across all studies for the same intervention.

^b^PACE: problem identification, alternative solution generation, consequences of each alternative solution, execute solution and evaluate.

^c^CBT: cognitive behavioral therapy.

^d^iCBT: internet-based cognitive behavioral therapy.

^e^FGID: functional gastrointestinal disorder.

^f^IBD: inflammatory bowel disease.

All except two were human-assisted (10/12, 83%); human-assisted interventions allowed patients to connect with research or psychosocial staff (ie, psychologist, psychology trainee, nurse, peer counselor) for support via emails, texts, phone calls, private online messaging centers, or message boards (Multimedia Appendix 3). Half of the interventions (6/12, 50%) had some element of caregiver involvement [45-47,52,55-58,61]. Skills practice was notably the only component shared across all interventions. Other components incorporated that lend themselves well to digital intervention platforms include ecological momentary assessment, which allows symptom tracking in real time (4/12, 33%) [64]; tailoring of content to individual users (6/12, 50%); didactic videos (4/12, 33%); online discussion forums (6/12, 50%); and gamification to increase engagement (3/12, 25%).

### Participants and Study Characteristics

Participant ages ranged from 10 to 18 years. The targeted chronic illnesses included diabetes, chronic pain, juvenile arthritis, cancer, chronic fatigue syndrome, recurrent headache, chronic respiratory condition, sickle cell disease, and gastrointestinal disorders (Table 1).

Of the 16 included efficacy studies (Tables 2 and 3), there were 3 single-site prospective cohort studies [44,53,57], 1 multisite prospective cohort study [52], 5 pilot RCTs (3 single-site [46,48,58], 2 multisite [49,60]), and 7 phase 2-3 RCTs (4 single-site [45,46,54,56,61], 3 multisite [47,55,59]). The 4 non-RCT prospective cohort studies were pilot studies with small sample sizes ranging from 2061 to 4049 participants with no comparison groups. For the pilot RCTs, sample sizes ranged from 1853 to 8357; one was described as powered for between-groups analyses [57]. The majority had an active treatment comparison condition, with only one using a wait-list control [56]. For the RCTs, sample sizes ranged from 4856 to 32,059; five were described as powered for between-groups analyses [45,55,56,59,61]. A little over half (4/7, 57%) [45,53-55,57,59,61] were compared with an active treatment condition, and the rest were compared with a wait-list control group.

Measures of psychosocial outcomes were collected across all studies, and physical health outcomes were collected in 88% (14/16) of studies [45-47,49,52-61] (Tables 2 and 3). However, outcomes assessed were heterogeneous, which prevents holistic synthesis across studies. At posttreatment, 56% (9/16) of the reviewed studies reported significant improvements in psychosocial outcomes (eg, anxiety, depression) [46,48,49,55,58,61]; effect sizes, where reported, ranged from small to large for RCTs and non-RCTs alike [46-49,55,58,61]. At posttreatment, half (8/16, 50%) [45-47,49,52,53,56] of the reviewed studies reported significant improvements in health-related outcomes (eg, physical functioning, disease knowledge); effect sizes, where reported, ranged from small to large for RCTs and non-RCTs alike [46,47,49,52,55,56,61]. In combination, a majority (11/16, 69%) of included studies reported some evidence of efficacy on psychosocial outcomes and/or health-related outcomes at posttreatment [45-49,52,53,55,56,58,59]. Findings across RCTs and non-RCTs were similar, with the exception that all non-RCTs reported some improvements in psychosocial outcomes.

**Table 2:** Original research publications with quantitative outcomes: randomized controlled trials only.

Intervention name		Type of study		Control group		Sample size		Powered for analyses?		Posttreatment outcomes^a.b^		Longitudinal outcomes^a.b^
Breathe Easier Online [44]		Pilot RCT^c^		Wait-list control		42				Psychosocial outcomes: no significant results for depression or social problem solving		N/A^d^
Fatigue in Teenagers on the InterNET [45]		RCT		Usual care		135		x		Psychosocial outcomes: intervention improved school attendance (*P*≤.01, 95% CI 2.7 to 8.9) Physical health outcomes: intervention improved fatigue (*P*≤.01, 95% CI 2.1 to 4.9) and physical functioning (*P*≤.01, 95% CI 2.3 to 6.3)		Psychosocial outcomes: intervention improved school attendance at 12 months Physical health outcomes: intervention improved fatigue and physical functioning at 12 months
iCanCope [60]		Pilot multisite RCT		iCanCope version A (symptom tracking only)		59				Psychosocial outcomes: no significant results for mood Physical health outcomes: no significant results for pain intensity and interference, physical activity, sleep quality, or energy		N/A
ICBT^e^ for adolescents with FGID^f^ [61]		RCT		Wait-list control		101		x		Psychosocial outcomes: intervention improved fear and worry about symptoms (95% CI 0.39 to 1.09, *d*^g^=0.74), and anxiety sensitivity (95% CI –0.07 to 0.47, *d*=0.20) No significant results for perceived stress or depressive symptoms Physical health outcomes: intervention improved gastrointestinal symptoms (95% CI 0.16 to 0.84, *d*=0.50) and pain intensity and frequency (95% CI 0.11 to 0.61, *d*=0.36)		Psychosocial outcomes: intervention improved fear and worry about symptoms (95% CI 0.59 to 1.59, *d*=1.05) and anxiety sensitivity (95% CI 0.10 to 1.04, *d*=0.57) at 6 months No significant results for perceived stress (95% CI –0.10 to 0.73, *d*=0.31) or depressive symptoms (95% CI –0.14 to 0.46, *d*=0.16) at 6 months Physical health outcomes: intervention improved gastrointestinal symptoms (95% CI 0.24 to 1.02, *d*=0.63) and pain intensity and frequency (95% CI 0.41 to 1.12, *d*=0.76) at 6 months
Move It Now [47]		Multisite RCT		Wait-list control		69				Psychosocial outcomes: intervention improved rewarding pain behavior by parents (*P*≤.01) and quality of life (*P*≤.01 to .04, *d*=–0.87 to 0.34) Physical health outcomes: intervention improved pain intensity (*P*=.03, *d*=–0.42), pain interference (*P*=.03, *d*=–0.46) and sleep problems (*P*≤.01, *d*=–0.60)		Psychosocial outcomes: intervention improved quality of life (besides mental health subdomain) at 3 months No significant results for rewarding pain behavior by parents at 3 months Physical health outcomes: no significant results for pain intensity, pain interference, or sleep problems at 3 months
TeenCope [59]		Multisite RCT		eHealth managing diabetes psychoeducation for self-management		320		x		Psychosocial outcomes: no significant results for quality of life, social acceptance, self-efficacy, perceived stress, or diabetes family conflict Physical health outcomes: no significant results for HbA_1c_^h^		Psychosocial outcomes: no significant results for quality of life, social acceptance, self-efficacy, perceived stress, or diabetes family conflict at 18 months Physical health outcomes: no significant results for HbA_1c_ at 18 months
Teens Taking Charge: Managing Arthritis Online [52]		Pilot multisite RCT		Attentional control		46				Psychosocial outcomes: no significant results for quality of life, self-efficacy, or stress Physical health outcomes: intervention improved disease knowledge (*P*≤.01, *d*=1.32) and pain intensity (*P*=.03, *d*=0.78)		N/A
Trautmann self-help programs: internet-based CBT, internet-based applied relaxation [54]		3-arm RCT		Internet psychoeducation intervention		65				Psychosocial outcomes: no significant results for pain catastrophizing or psychological well-being Physical health outcomes: no significant results for headache frequency and duration		Psychosocial outcomes: no significant results for pain catastrophizing or psychological well-being at 6 months Physical health outcomes: no significant results for headache frequency and duration at 6 months
Trautmann’s internet-based CBT [53]		Pilot RCT		Internet psychoeducation intervention		18				Psychosocial outcomes: intervention improved pain catastrophizing (*P*≤.05) Physical health outcomes: intervention improved headache frequency (*P*≤.05) No significant results for headache intensity or headache duration		Psychosocial outcomes: intervention improved pain catastrophizing (*P*≤.05) at 6 months Physical health outcomes: intervention improved headache frequency (*P*≤.05) at 6 months No significant results for headache intensity or headache duration at 6 months
Web-based management of adolescent pain												
	Multisite RCT [55]		Internet education control		273		x		Psychosocial outcomes: intervention improved emotional functioning (*P*=.04, *d*=–0.09) Physical health outcomes: no significant results for activity limitations, pain intensity, or sleep quality		Psychosocial outcomes: no significant results for emotional functioning at 6 months Physical health outcomes: intervention improved activity limitations (*P*=.03; *d*=–0.25) and sleep quality (*P*=.04, *d*=0.16) at 6 months No significant results for pain intensity at 6 months	
	RCT [56]		Wait-list control		48		x		Psychosocial outcomes: no significant results for depression or parental protectiveness Physical health outcomes: intervention improved activity limitations (*P*≤.01, η^2i^=.17) and pain intensity (*P*=.03, η^2^=.11)		Psychosocial outcomes: no significant results for depression and parental protectiveness at 3 months Physical health outcomes: intervention improved activity limitations and pain intensity at 3 months	
	Pilot RCT [57]		Specialized headache treatment		83		x		Psychosocial outcomes: no significant results for anxiety or depression Physical health outcomes: no significant results for headache frequency, pain intensity, activity limitations, sleep duration, or sleep efficiency		Psychosocial outcomes: no significant results for anxiety or depression at 3 months Physical health outcomes: no significant results for headache frequency, pain intensity, activity limitations, sleep duration, or sleep efficiency at 3 months	

^a^Only analyses of between-group differences comparing the intervention and control arms are reported.

^b^Information regarding confidence intervals, effect size, and *P* values is included when reported in the original research publication.

^c^RCT: randomized controlled trial.

^d^N/A: not applicable.

^e^iCBT: internet-based cognitive behavioral therapy.

^f^FGID: functional gastrointestinal disorder.

^g^*d*: Cohen *d*.

^h^HbA_1c_: hemoglobin A_1c_.

^i^η^2^: eta squared.

**Table 3:** Original research publications with quantitative outcomes: nonrandomized controlled trials.

Intervention name	Type of study	Sample size	Posttreatment outcomes^a^	Longitudinal outcomes^a^
ICBT^b^ for adolescents with FGID^c^ [46]	Pilot study	29	Psychosocial outcomes: intervention improved stress (*P*<.05, 95% CI 0.02 to 0.69, *d*^d^=0.35) at posttreatment No significant results for anxiety or depression at posttreatment Physical health outcomes: intervention improved gastrointestinal symptoms (*P*<.05, 95% CI 2.37 to 10.58, *d*=0.50), pain interference (*P*<.05, 95% CI 0.11 to 0.61, *d*=0.36), and pain reactivity (*P*<.05, 95% CI 0.39 to 1.09, *d*=0.74) at posttreatment No significant results for functional disability at posttreatment	Psychosocial outcomes: intervention improved anxiety (*P*<.05, 95% CI 0.08 to 0.81, *d*=0.44) at 6 months No significant results for depression or stress at 6 months Physical health outcomes: intervention improved gastrointestinal symptoms (95% CI 3.43 to 12.21, *d*=0.63), pain interference (*P*<.05, 95% CI 0.41 to 1.12, *d*=0.76), pain reactivity (*P*<.05, 95% CI 0.59 to 1.59, *d*=1.05), and functional disability (*P*<.05, 95% CI 0.10 to 1.04, *d*=0.56) at 6 months
PainSquad+ [49]	Multisite pilot study	40	Psychosocial outcomes: intervention improved emotional functioning (*P*≤.01, *d*=0.66), social functioning (*P*≤.01, *d*=0.46), and overall HRQOL^e^ (*P*=.02, *d*=0.43) at posttreatment No significant results for self-efficacy, or school functioning at posttreatment Physical health outcomes: intervention improved pain intensity (*P*≤.01, *d*=0.67) and pain interference (*P*=.03, *d*=0.38) at posttreatment No significant results for physical functioning at posttreatment	N/A
Onco-STEP [48]	Pilot study	20	Psychosocial outcomes: intervention improved posttraumatic stress symptoms (*P*≤.01, *d*=0.63), anxiety (*P*≤.01, *d­*=0.74), fear of progression/relapse (*P*<.05, *d*=0.48), and depression (*P*≤.01, *d*=1.0) at posttreatment	Psychosocial outcomes: intervention improved posttraumatic stress symptoms (*P*<.01), fear of progression/relapse (*P*<.01), and anxiety (*P*<.01) at 3 months No significant results for depression at 3 months
Web-based treatment for adolescents with IBD^f^ [58]	Pilot study	24	Psychosocial outcomes: intervention improved somatic symptoms (*P*≤.01, η^2g^=.41), approach coping strategies (*P*≤.01, η^2^=.43), distraction techniques (*P*≤.01, η^2^=.35), protective parenting behaviors (*P*=.03, η^2^=.27) at posttreatment Physical health outcomes: no significant results for abdominal pain at posttreatment	Psychosocial outcomes: intervention improved protective parenting behaviors (*P*=.01, η^2^=.44) at 6 months No significant results for somatic symptoms, approach coping strategies, or distraction techniques at 6 months Physical health outcomes: no significant results for abdominal pain at 6 months

^a^ICBT: internet-based cognitive behavioral therapy.

^b^Information regarding confidence intervals, effect size, and *P* values are included when reported in the original research publication.

^c^FGID: functional gastrointestinal disorder.

^d^*d*: Cohen *d*.

^e^HRQOL: health-related quality of life.

^f^IBD: inflammatory bowel disease.

^g^η^2^: eta squared.

A subset of 75% (12/16) of studies [45-48,53-59,61] evaluated longer term assessment time points ranging from 3 to 18 months (Tables 2 and 3). Nine studies (9/16, 56%) [45-48,53,55,56,58,61] showed promise of longer term gains from the intervention.

### Risk of Bias

**Figure 2:**
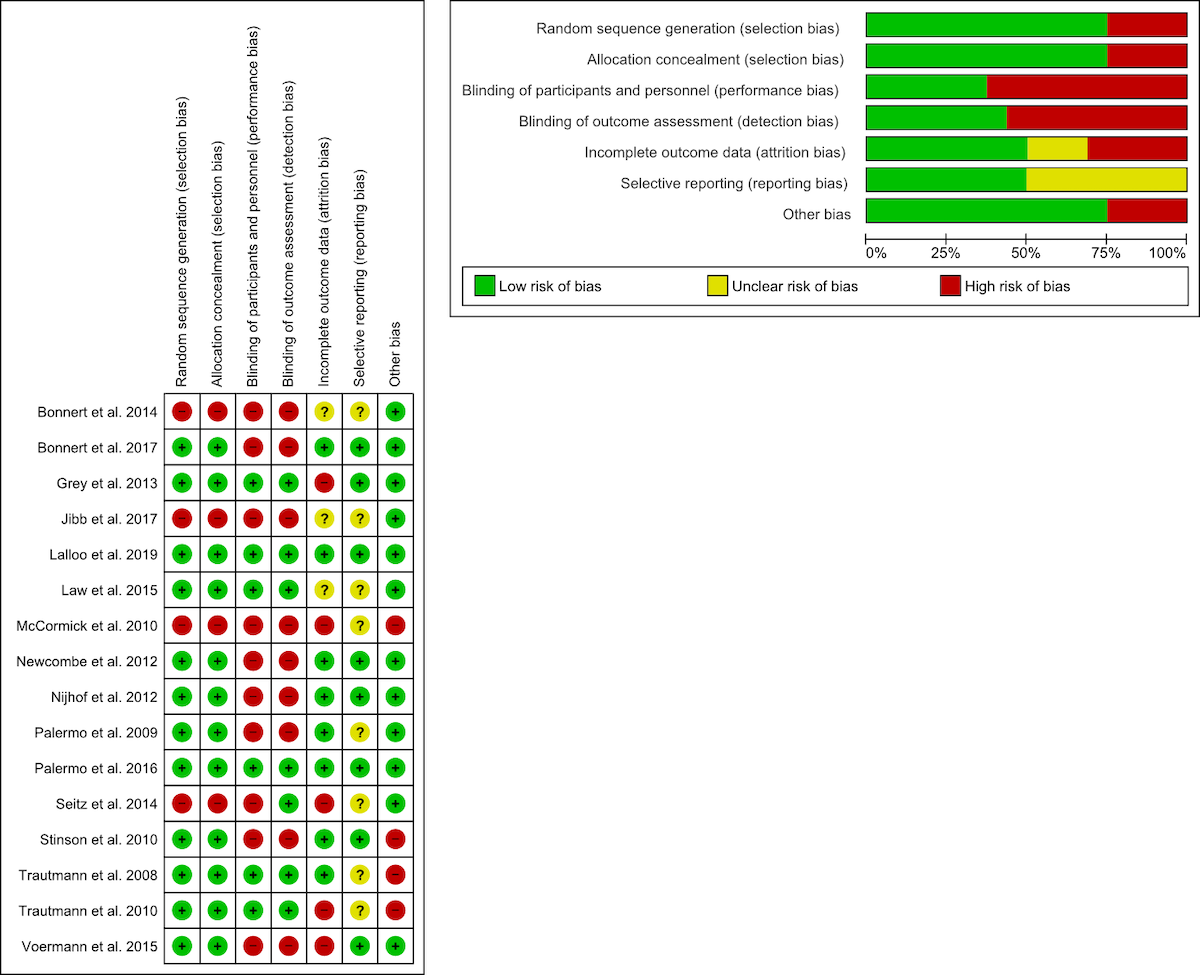
Risk of bias.

Risk of bias was evaluated for all included studies (Figure 2). Of the 16 studies, 12 reported random sequence generation and allocation concealment (ie, the pilot RCTs and RCTs). For the blinding of participants and personnel domain, 10 were high risk and 6 low risk; high-risk studies consisted of study designs with no control group or a wait-list control group. For the blinding of outcome assessment domain, 9 were high risk and 7 low risk. For attrition bias, 8 were low risk, 5 high risk, and 3 unclear; note that attrition rates for internet-based interventions (with an anchor point of around 50%) tend to be higher than traditional face-to-face psychosocial interventions [65]. For selective reporting bias, studies were split in half between low risk and unclear; studies were rated as unclear due to a lack of clinical trial registration or published protocol. For other biases, 12 were considered low risk and 4 high risk.

## Discussion

### Overview

A majority of youth with chronic illnesses struggle with issues that psychosocial interventions effectively manage such as anxiety, stress, depression, maladjustment, pain, and poor coping skills [4,7]. Within the past decade, digital health interventions have been increasingly popular with widespread access to the internet and smartphones. To our knowledge, this is the first systematic review summarizing the research evidence in support of the efficacy of eHealth and mHealth psychosocial interventions for youth chronic illnesses. Each of the interventions identified was designed for a specific chronic illness population. The state of the science is still in its nascent stages, with only 16 published efficacy studies of 12 unique interventions identified. We provided information on the structure and content of included interventions and relationships between each of the interventions and various psychosocial and health outcomes. Given the substantial number of studies in which full articles were reviewed for eligibility (n=133, Figure 1), this points to growing interest in digital interventions for youth with chronic illnesses. Few digital interventions have advanced to the stages of efficacy testing (n=16), and even fewer in an RCT with an active comparison condition (n=8). This systematic review suggests that disseminating traditional evidence-based psychotherapies via novel digital health technologies may be efficacious.

Consistent with evidence-based digital health practices and recent systematic reviews of digital interventions with youth chronic illness and mental health populations [17,24,25,28,33], our systematic review of eHealth and mHealth psychosocial interventions for youth chronic illness populations found (a) early evidence of improvements in psychosocial and physical health outcomes immediately posttreatment; (b) early evidence of the maintenance of treatment gains at longer term follow-up extending to 3+ months; (c) a prevalence of computerized cognitive behavioral therapy interventions; (d) varying levels of psychosocial staff support via text, email, phone, online discussion forums, or private online messaging centers; and (e) key methodological limitations for a majority of included studies such as lack of blinded outcomes assessment, limited number of RCTs, and few studies with active treatment comparison groups.

This review also suggests several gaps in the existing literature. Future research should focus on optimizing digital intervention design and implementation, namely how to efficiently streamline resource-intensive personnel assistance and encourage self-direction in order to sustain intervention efficacy and engagement while minimizing costs [66]. Notably, only two of the included interventions did not involve some form of contact with providers or research staff. In addition to clinician involvement, given that half of the interventions reviewed included caregiver support, new digital intervention research should continue to explore the additive value of caregiver involvement where appropriate with unique content designed for parent caregivers [55,56]. Next, as each of the interventions included in our review was designed for a specific chronic illness, further research is needed to ascertain whether a disease nonspecific transdiagnostic approach [67,68] to designing digital psychosocial interventions applicable across heterogeneous disease groups is warranted. In particular, online mindfulness-based interventions have been shown to be successful in chronic illness and other populations [69-71]. Interventions also had multiple components, which may benefit from dismantling studies to identify which active therapeutic ingredients lead to positive outcomes [72]. Similarly, emphasis should be placed on identifying and targeting the aspects of digital engagement that lead to the intended behavior change rather than just encouraging more frequent use [73]. The use of analytics in eHealth and mHealth can provide invaluable insights into active therapeutic ingredients, aspects of effective digital engagement, treatment moderators (for whom interventions work), and treatment mediators (how treatments work) [74,75]. Finally, future studies might assess the value of more novel technologically enabled features such as just-in-time adaptive intervention designs (JITAIs). Indeed, JITAIs use causal modeling to identify the appropriate type and dose of intervention at the optimal point in time by measuring and responding to an individual’s changing health states [76,77]. These innovative programs have the potential to meet evolving real-time needs of youth at risk.

Given the interest in and rapidly changing landscape of digital health, it is likely that new publications have been released during the typical time frame it had taken us to rigorously complete our systematic review. We intend to publish a 5-year systematic review update populated with new efficacy studies and trials. The median timing of Cochrane systematic review updates is greater than 5 years [78]. Although previous research suggests only 4% of systematic review updates report a change in conclusions [79], our overarching findings may be subject to change given the limited number of included studies and the quickly evolving digital health landscape.

### Limitations

Limitations to consider are as follows. First, most of the interventions were designed and tested as web-based eHealth interventions, with only two mHealth intervention designed for use on smartphone devices. We anticipate that more mHealth interventions will be tested for efficacy and come down the pipeline in the years to come as smartphones become more and more ubiquitous. Previous research suggests that mHealth apps are widely accepted by the general public for coping skills and stress management, and beliefs and attitudes toward mHealth are positive [80]. Second, the majority of studies were not powered to detect meaningful changes in health outcomes of interest nor did they designate primary versus secondary outcomes in their research designs. Third, only half of the studies used an active treatment comparison condition or blinded participants to assigned treatment condition, and findings of favorable treatment response may be susceptible to the placebo effect [81]. Fourth, there exists the possibility of publication bias because nonsignificant findings are often difficult to publish. Fifth, although our search was executed by a medical librarian in five well-established library databases, other databases to which our university does not have access (eg, Scopus) may have uncovered additional relevant publications. Finally, given the heterogeneity of disease groups, measurement time points, and study outcomes measures, it was not possible to conduct a meta-analysis and provide synthesized results of the efficacy studies included in our systematic review.

### Conclusions

The strengths of this paper include the systematic approach to synthesizing the great breadth of literature across pediatric illness populations and the eHealth and mHealth focus increasingly popular among youths. This publication is especially timely given heightened psychological distress and exacerbating mental health symptoms for youth in the context of the COVID-19 pandemic, in-home confinement, school closures, social distancing, and a shift toward online and telehealth services [82-85]. We found intervention features unique to digital platforms such as gamification, ecological momentary assessment, and machine learning algorithms. Such features capitalize on technological advances to intervene during distressing situations in real time and tailor content to individual preferences and needs. Leveraging such technological advances allows movement toward a data-driven and personalized approach to precision mental health [86]. The state of the science is still in its early stages, and further clinical trials research is needed to confirm whether evidence-based psychosocial interventions traditionally delivered in-person may be successfully translated to digital formats for a range of youth chronic illness populations.

## Appendix

Multimedia Appendix 1 Primary search strategy.

Multimedia Appendix 2 Supplemental search strategy.

Multimedia Appendix 3 Intervention and personnel-supported components.

## Abbreviations

JITAI: just-in-time adaptive intervention design
mHealth: mobile health apps
PRISMA: preferred reporting items for systematic reviews and meta-analyses
RCT: randomized controlled trial
