# Supplementary material for: eHealth and mHealth Psychosocial Interventions for Youths With Chronic Illnesses: Systematic Review
Source: JMIR Pediatr Parent. 2020 Nov 10;3(2):e22329. doi: 10.2196/22329 (PMC7685926; doi:10.2196/22329)
Supplement: Multimedia Appendix 1 [file pediatrics_v3i2e22329_app1.docx]

**Multimedia Appendix 1.** Primary search strategy.

| **Search Term 1^a^** | **Search Term 2** | **Search Term 3** |
| --- | --- | --- |
| Mobile applications | Malignant neoplasm | Children |
| Internet | Neoplasms subdivided by anatomical site | Adolescents |
| mHealth | Cancer patient | Young adults |
| eHealth | Cancer | Pediatric* |
| Telemedicine | Neoplasm | Teen* |
| Telehealth | Malignan* NEAR tumor* | Preteen* |
| Smartphone | Chronic disease | Adolescen* |
| Smartphone devices | Multiple chronic conditions | Preadolescen* |
| Smartphone application | Chronic patient | Youth* |
| iPhone | Multimorbidit* | Young adult* |
| Internet | Chronic OR noncommunicable  OR long term NEAR condition* or disease* OR ill* OR sick* OR syndrom* | Juvenile |
| Online | Palliative therapy | School* |
| Mobile software | Palliat* | Student* OR Age* NEAR middle school OR junior high OR high school OR college OR university |
| Portable software |  | Generation NEAR Y OR Z OR millenni* OR boomlet |
| App * |  |  |
| Application* |  |  |

Total number of results: 3,150 (Medline: 1,350, Embase: 778, PsycINFO: 573, Web of Science: 371, Cochrane Database of Systematic Reviews: 78)

Filter: 2008-2019, English language.

^a^ Search terms in each column/category were combined using Boolean operators
