# Supplementary material for: eHealth and mHealth Psychosocial Interventions for Youths With Chronic Illnesses: Systematic Review
Source: JMIR Pediatr Parent. 2020 Nov 10;3(2):e22329. doi: 10.2196/22329 (PMC7685926; doi:10.2196/22329)
Supplement: Multimedia Appendix 2 [file pediatrics_v3i2e22329_app2.docx]

**Multimedia Appendix 2.** Supplemental search strategy.

| **Search Term 1^a^** | **Search Term 2** | **Search Term 3** | **Search Term 4** |
| --- | --- | --- | --- |
| Internet-deliver* | Diabet* | Systematic review | Intervention |
| eHealth | Functional gastrointestinal OR FGD OR Functional GI | Systematic literature review |  |
| mHealth | Irritable bowel syndrome OR IBS OR inflammatory bowel disease OR IBD | Metaanal* |  |
| Mobile health | Chronic Fatigue Syndrome OR CFS OR Systemic Exertion Intolerance Disease OR SEID OR myalgic encephalomyelitis | Metasynth* |  |
| Mobile app* | Arthriti* OR Osteoarthriti* |  |  |
| Mobile phone app* | Respirat* |  |  |
| Smartphone | Asthma |  |  |
| Smart phone | Sickle cell |  |  |
|  | Traumatic brain injury OR traumatic brain injuries OR TBI |  |  |
|  | Skin or Skin condition* or Skin disease* or eczema |  |  |

Total number of results: 701 (PubMed: 253, MedLine & PsycINFO: 266, Web of Science: 182)

Filter: 2008-2019, English language.

^a^ Search terms in each column/category were combined using Boolean operators.
