# Supplementary material for: eHealth and mHealth Psychosocial Interventions for Youths With Chronic Illnesses: Systematic Review
Source: JMIR Pediatr Parent. 2020 Nov 10;3(2):e22329. doi: 10.2196/22329 (PMC7685926; doi:10.2196/22329)
Supplement: Multimedia Appendix 3 [file pediatrics_v3i2e22329_app3.docx]

Multimedia Appendix 2. Intervention and personnel-supported components.

| Intervention name | Intervention components | | | | | | | | | | | | Personnel supported components | | |
| --- | --- | --- | --- | --- | --- | --- | --- | --- | --- | --- | --- | --- | --- | --- | --- |
|  | Gamification | Rewards system^a^ | Videos | Personalization | Machine learning | Skills practice | Motivational messages | Reminders/Notifications | Symptom monitoring | Peer support/ discussion board | Journaling | Parental involvement | Personnel-assisted | Support provided by | Mode of communication |
|  |  |  |  |  |  |  |  |  |  |  |  |  |  |  |  |
| Breathe Easier Online [44] |  |  |  | x |  | x |  |  |  | x |  |  | x | Research staff | Private online message center |
| Fatigue in Teenagers on the InterNET [45] |  |  |  |  |  | x |  |  |  |  | x | x | x | Cognitive behavioral psychotherapist | Email (phone in case of emergency) |
| iCanCope [60] |  |  |  |  |  | x | x | x | x | x |  |  |  | — | — |
| ICBT^b^ for adolescents with FGID^c^ [46,61] |  |  |  |  |  | x | x |  |  |  |  | x | x | Psychologist, PhD clinical psychology student | Email on internet platform, phone |
| Move It Now [47] |  |  |  | x |  | x |  |  |  |  |  | x | x | Therapist^d^ | Email, phone |
| Onco-STEP [48] |  |  |  |  |  | x |  |  |  |  | x |  | x | Clinical psychologist | Web-based text messages, phone |
| PainSquad+ [49,50] | x | x | x | x | x | x |  | x | x | x |  |  | x | Registered nurse | Phone |
| TeenCope [51] | x |  | x | x |  | x |  | x |  | x |  |  |  | — | — |
| Teens Taking Charge: Managing Arthritis Online [52] |  |  | x | x |  | x |  |  |  | x | x | x | x | Trained coach (nonhealth care professional with undergraduate degree in psychology) | Phone, email, online discussion board |
| Trautmann self-help programs: internet-based CBT, internet-based applied relaxation [53,54] |  |  |  |  |  | x |  | x | x |  |  |  | x | Clinical psychology trainees (PhD candidates) | Email, phone |
| Web-based management of adolescent pain [55-57] | x |  | x | x |  | x | x | x | x |  |  | x | x | Clinical psychology trainees (master’s level or PhD postdoctoral fellows) | Private online message center |
| Web-based treatment for adolescents with IBD^e^ [58] |  |  |  |  |  | x | x |  |  | x |  | x | x | Research staff | Private online message center |

^a^Although rewards systems can be subsumed under digital gamification, we have listed this separately as rewards systems are a common element in face-to-face pediatric psychosocial interventions.

^b^iCBT: internet-based cognitive behavioral therapy.

^c^FGID: functional gastrointestinal disorder.

^d^Language used to describe providers taken directly from article.

^e^IBD: irritable bowel disease.
